# The cyclin-dependent kinase inhibitor AT7519 accelerates neutrophil apoptosis in sepsis-related acute respiratory distress syndrome

**DOI:** 10.1136/thoraxjnl-2016-209229

**Published:** 2016-10-24

**Authors:** David A Dorward, Jennifer M Felton, Calum T Robb, Thomas Craven, Tiina Kipari, Timothy S Walsh, Christopher Haslett, Kallirroi Kefala, Adriano G Rossi, Christopher D Lucas

**Affiliations:** 1 The MRC Centre for Inflammation Research, Queen's Medical Research Institute, University of Edinburgh, Edinburgh, UK; 2 Department of Critical Care, Anaesthesia and Pain Medicine, Royal Infirmary of Edinburgh, Edinburgh, UK

**Keywords:** Neutrophil Biology, ARDS, Innate Immunity

## Abstract

Acute respiratory distress syndrome (ARDS) is a neutrophil-dominant disorder with no effective pharmacological therapies. While the cyclin-dependent kinase inhibitor AT7519 induces neutrophil apoptosis to promote inflammation resolution in preclinical models of lung inflammation, its potential efficacy in ARDS has not been examined. Untreated peripheral blood sepsis-related ARDS neutrophils demonstrated prolonged survival after 20 hours *in vitro* culture. AT7519 was able to override this phenotype to induce apoptosis in ARDS neutrophils with reduced expression of the pro-survival protein Mcl-1. We demonstrate the first pharmacological compound to induce neutrophil apoptosis in sepsis-related ARDS, highlighting cyclin-dependent kinase inhibitors as potential novel therapeutic agents.

## Introduction

Acute respiratory distress syndrome (ARDS) is a neutrophil-dominant disease with significant morbidity and mortality but no effective pharmacological therapies exist. Pulmonary neutrophil accumulation results in release of chemokines, proteases and reactive oxygen species perpetuating inflammation and tissue injury. Augmenting neutrophil apoptosis in order to accelerate the resolution of inflammation has been proposed as a treatment strategy.^1–3^

Neutrophils are short-lived granulocytes that undergo energy-dependent and caspase-dependent programmed cell death (apoptosis) within hours. Apoptosis, in concert with clearance by phagocytes, results in a pro-resolution phenotype restoring tissue homeostasis. Neutrophil survival is governed by both pro-apoptotic and anti-apoptotic factors in the intracellular and extracellular environment. Granulocyte macrophage-colony stimulating factor (GM-CSF), tumour necrosis factor (TNF), hypoxia and bacterial endotoxins increase lifespan partly through upregulation of intracellular proteins including the Bcl-2 family member Mcl-1. Spontaneous apoptosis is therefore delayed in various diseases including ARDS, cystic fibrosis and sepsis and is thought to contribute to disease pathogenesis.^3–5^

Apoptosis in neutrophils isolated from healthy human volunteers has been achieved *in vitro* by phosphoinositide 3-kinase (PI3K) and cyclin-dependent kinase (CDK) inhibitors, polyphenolic flavones and lipid mediators.^6^ We first described CDK inhibitor (CDKi)-induced neutrophil apoptosis^1^ and have subsequently demonstrated its mechanistic function through CDK7 and CDK9-mediated alterations in Mcl-1.^2^ ^7^ Importantly, CDKi can override multiple pro-survival factors *in vitro* including GM-CSF and bacterial endotoxins. The CDKi AT7519 potently inhibits CDK9 and, at higher concentrations, other CDKs including CDK2 and CDK5 but not non-CDK kinases^8^ driving neutrophil apoptosis and subsequent resolution of inflammation in multiple models of pulmonary inflammation.^2^ We therefore hypothesised that AT7519 would override delayed neutrophil apoptosis in ARDS providing further evidence of its potential therapeutic value.

## Methods

Venous blood was collected from mechanically ventilated patients with ARDS (defined according to Berlin criteria)^9^ and age-matched and sex-matched healthy controls (see online supplementary table S1). Written, informed consent was obtained (from the next of kin in cases of incapacity). Approval was from the Lothian Research Ethics Committee (13/SS/0157, 15-HV-013).

10.1136/thoraxjnl-2016-209229.supp1 supplementary table Demographic and clinical data for ARDS patients and control subjects

Neutrophils, isolated by dextran sedimentation and Percoll gradient, were cultured in Iscove's modified Dulbecco's medium (Gibco) (5×10^6^ cells/mL; 5% autologous or fetal calf serum) in the presence or absence of AT7519 (Astex Pharmaceuticals). Neutrophil apoptosis was examined by flow cytometry (Annexin-V (Roche) and propidium iodide (Sigma)) and confirmed by cytocentrifuge and Diff-Quik staining (Gamidor).^10^ Mcl-1, cleaved caspase-3 and β-actin expressions were determined by western blotting.^10^ Plasma C reactive protein (CRP) and GM-CSF were measured by ELISA (RnD Systems), while TNF, interleukin (IL)-1β, IL-6, IL-8, IL-10 and IL-12p70 were quantified by cytokine bead array (BD Bioscience).

Flow cytometry data were analysed using FlowJo 10.0.8 (TreeStar) and statistical analyses with Prismv7 (GraphPad); significance was accepted at p<0.05.

## Results

**Figure 1:**
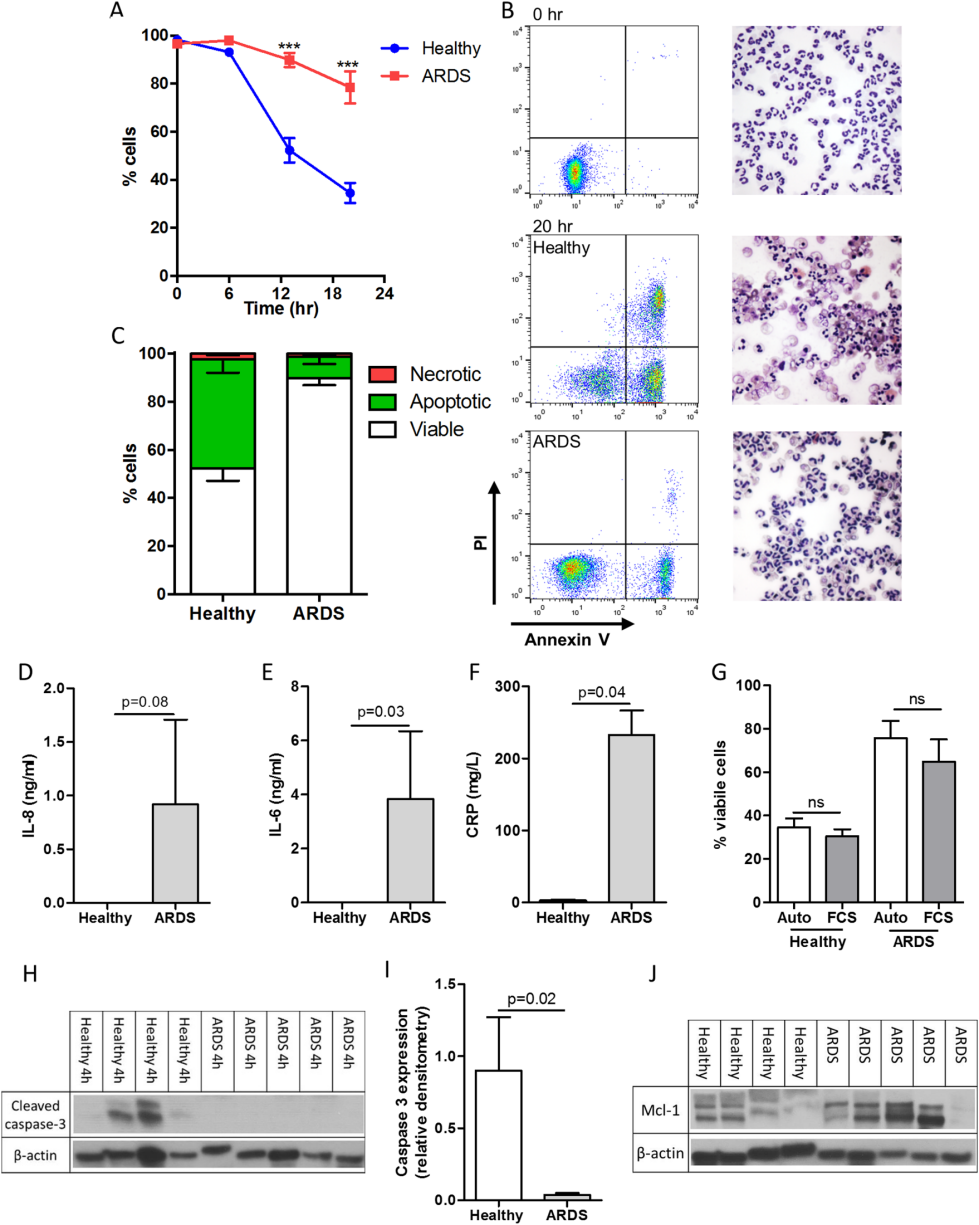
Neutrophils isolated from patients with acute respiratory distress syndrome (ARDS) exhibit an intrinsic delay in spontaneous apoptosis. Blood neutrophils from patients with ARDS and healthy volunteers were cultured for 0, 6, 13 and 20 hours with cell viability (AnnV−ve/PI−ve), apoptosis (AnnV+ve/PI−ve) and necrosis (PI+ve) assessed by flow cytometry. (A) Cell viability over the time period; (B) representative flow cytometry plots and cytocentrifuge preparations at 0 and 20 hours (400× magnification); (C) the proportion of viable, apoptotic and neutrophils following 13 hours culture; circulating interleukin (IL)-8 (D), IL-6 (E) and C reactive protein (CRP) (F) levels in plasma; (G) cell viability of both healthy control and ARDS neutrophils at 20 hours following incubation with autologous (Auto) or fetal calf serum (FCS); (H) cleaved caspase 3 expression following 4 hours culture quantified by densitometry (I), (J) Mcl-1 expression in freshly isolated neutrophils are shown. (A) ***p<0.001 repeated measures analysis of variance with Sidak's multiple comparisons test, (D)–(G) and (I) Mann-Whitney U test ((A) (C) and (G)) n=5/group; (D)–(F) n=3 healthy, n=5 ARDS; (H)–(J) n=4 healthy, n=5 ARDS)).

Unstimulated peripheral blood neutrophils from patients with ARDS had greater survival compared with age-matched and sex-matched healthy controls following 13 and 20 hours of culture (figure 1A). This enhanced viability was due to delayed spontaneous apoptosis (healthy 45.3% vs ARDS 8.9% at 13 hours (% total cells; p=0.008)) (figure 1B, C). IL-6 and CRP were increased in ARDS plasma with IL-8 detectable in all but one of the patients with ARDS and undetectable in all controls (figure 1D–F). No difference in GM-CSF was observed, while all other cytokines were below the detection limit (data not shown). To investigate if delayed apoptosis was neutrophil-intrinsic or required a serum-derived factor, cells were cultured in fetal calf serum or autologous serum. This did not alter viability/apoptosis at any point (20 hours data shown; figure 1G). In keeping with the apoptosis data, caspase-3 cleavage was seen in healthy neutrophils but not ARDS neutrophils by 4 hours confirming alterations in intracellular apoptotic proteins (figure 1H, I). Furthermore, there was a trend towards increased intracellular Mcl-1 in ARDS neutrophils at 0 hours but with variable expression in both groups (figure 1J).

**Figure 2:**
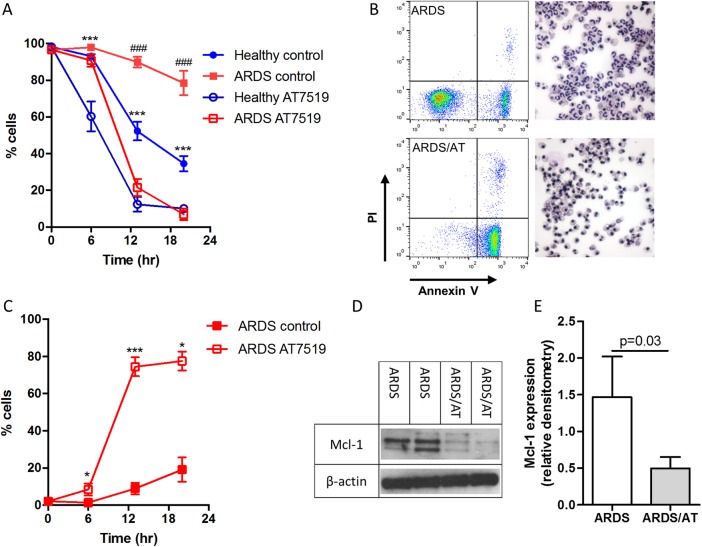
AT7519 induces time-dependent neutrophil apoptosis in acute respiratory distress syndrome (ARDS) with associated loss of Mcl-1 expression. Neutrophils were cultured in the presence or absence of the cyclin-dependent kinase inhibitor AT7519 (AT; 1 μM) for 0, 6, 13 and 20 hours with subsequent flow cytometry analysis. (A) Cell viability over the time period, (B) representative flow cytometry plots and cytocentrifuge preparations at 20 hours from ARDS neutrophils (400× magnification); (C) apoptosis of ARDS neutrophils following treatment with AT7519 over the time period; (D) representative paired Mcl-1 expression after 4 hours treatment with or without AT7519 (n=2) with cumulative densitometry (n=5) (E) shown. (A) and (C) *p<0.05, ***p<0.001 repeated measures analysis of variance with Sidak's multiple comparisons test; (A) *comparison between healthy volunteer neutrophil treatment groups, #comparison between ARDS neutrophils; (E) Wilcoxon matched-pairs signed-rank test; n=5/group all experiments.

AT7519 induced neutrophil apoptosis in healthy volunteer neutrophils within 6 hours. In ARDS neutrophils, AT7519 induced apoptosis but only after 13 hours of culture. By 20 hours apoptosis was at a level equivalent to AT7519-treated healthy control cells, thus completely overriding the pro-survival phenotype (figure 2A–C). Necrosis was limited in all treatment groups commensurate with apoptosis being the primary mechanism of cell death. In keeping with the mechanism of AT7519-induced apoptosis in healthy neutrophils,^2^ there was significant reduction in Mcl-1 expression by 4 hours in AT7519-treated ARDS neutrophils (figure 2D).

## Discussion

Within the highly complex proinflammatory milieu, increased neutrophil survival provides the opportunity for continued release of toxic mediators to exacerbate tissue injury and potentiate inflammation. Indeed, delayed neutrophil apoptosis correlates with disease severity in sepsis and associated lung injury,^5^ while accelerated apoptosis induces inflammation resolution in several preclinical models of injury.^1^ ^2^ ^7^ In keeping with previous research, our study demonstrates delayed spontaneous apoptosis in ARDS peripheral blood neutrophils. This effect is independent of continual exposure to extrinsic serum-derived factors which are capable of prolonging the lifespan of healthy control neutrophils such as Fas and granulocyte-CSF.^11^ ^12^ In this small cohort, there was a trend towards increased Mcl-1 expression in ARDS neutrophils; increased Mcl-1 expression has previously been described in severe sepsis,^13^ the underlying aetiology in all our study patients. Although detailed profiling of other Bcl-2 family members was not performed due to limited sample availability, Mcl-1 appears particularly relevant to neutrophil survival as alterations in Mcl-1 alone is sufficient to alter neutrophil apoptosis rates.^2^

Recent elegant phenotyping of peripheral blood and alveolar neutrophils in ARDS has been described.^3^ Despite promising preclinical data delineating the role of isoform-selective PI3K inhibition in overriding GM-CSF-induced neutrophil survival, PI3K inhibition was unable to induce apoptosis in ARDS blood neutrophils,^3^ thereby questioning its utility as a potential therapy in ARDS. Our previous work has shown that CDK inhibition downregulates Mcl-1 with subsequent caspase activation and neutrophil apoptosis in healthy human neutrophils and preclinical models of inflammation.^1^ ^2^ In contrast to PI3K inhibition, we observed that CDK inhibition with AT7519 induced ARDS neutrophil apoptosis while simultaneously downregulating Mcl-1. This ability of CDK inhibition to induce ARDS neutrophil apoptosis is, to our knowledge, the first pharmacological compound shown to be capable of overriding delayed neutrophil apoptosis in ARDS. Evaluation of the effect of CDK inhibition on transmigrated alveolar neutrophils is a logical future step but was not possible due to constraints on patient recruitment.

Taken together, this suggests that intrinsic, PI3K-independent factors act to delay neutrophil apoptosis in ARDS and supports the conclusion that Mcl-1 targeted therapies may be beneficial in human disease. CDKi-augmented neutrophil apoptosis may therefore enhance resolution of lung inflammation and serve as a potential therapeutic strategy in ARDS.
