# Supplementary material for: The cyclin-dependent kinase inhibitor AT7519 accelerates neutrophil apoptosis in sepsis-related acute respiratory distress syndrome
Source: Thorax. 2016 Oct 24;72(2):182–5. doi: 10.1136/thoraxjnl-2016-209229 (PMC5284332; doi:10.1136/thoraxjnl-2016-209229)
Supplement: supplementary table — Demographic and clinical data for ARDS patients and control subjects [file thoraxjnl-2016-209229supp_table.pdf]

Table S1 – Demographic and clinical data for ARDS patients and control subjects

	Control (n=5)	ARDS (n=5)	p value
Mean age (yrs) (range)	58.2 (40-64)	59.4 (41-68)	0.83[A]
Sex (M/F)	4/1	4/1	1.0[B]
Smoking (% current)	20	20	1.0[B]
Apache II score<br>Mean (95% CI)	--	21.8 (13.5-30.0)	
PaO_2/FiO_2 (mmHg)<br>Mean (95% CI)	--	157 (120-193)	
Sepsis (%)	--	100	
≥1 co-morbidity (%)	--	80[C]	
Mortality (%)[D]	--	20	

APACHE II Acute Physiology and Chronic Health Evaluation II, PaO_2 partial pressure arterial oxygen, FiO_2 fraction of inspired oxygen; [A]t-test; [B] chi-squared; [C] co-morbidities were chronic obstructive airways disease, coronary artery disease, chronic liver disease, chronic neurological illness (unspecified) and past history of treated leukaemia; [D] assessed at time of discharge from intensive care
